# Longitudinal trends in incidence and health care use for pediatric concussion in Alberta, Canada

**DOI:** 10.1038/s41390-022-02214-5

**Published:** 2022-09-09

**Authors:** Krystle Wittevrongel, Olesya Barrett, Isabelle Couloigner, Stefania Bertazzon, Brent Hagel, Kathryn J. Schneider, David Johnson, Keith O. Yeates, Jennifer D. Zwicker

**Affiliations:** 1 School of Public Policy, University of Calgary, Calgary, AB Canada; 2 Alberta Health Services, Calgary, AB Canada; 3 Department of Geography, University of Calgary, Calgary, AB Canada; 4 O’Brien Institute for Public Health, University of Calgary, Calgary, AB Canada; 5 Department of Pediatrics, Cumming School of Medicine, University of Calgary, Calgary, AB Canada; 6 Department of Community Health Sciences, Cumming School of Medicine, University of Calgary, Calgary, AB Canada; 7 Alberta Children’s Hospital Research Institute, University of Calgary, Calgary, AB Canada; 8 Sport Injury Prevention Research Centre, Faculty of Kinesiology, University of Calgary, Calgary, AB Canada; 9 Faculty of Kinesiology, University of Calgary, Calgary, AB Canada; 10 Hotchkiss Brain Institute, University of Calgary, Calgary, AB Canada; 11 Department of Clinical Neurosciences, Cumming School of Medicine, University of Calgary, Calgary, AB Canada; 12 Department of Psychology, University of Calgary, Calgary, AB Canada

## Abstract

**Background:**

We described longitudinal trends in the incidence of episodes of care (EOC) and follow-up care for pediatric concussion in relation to age, sex, rurality of patient residence, point of care, and area-based socioeconomic status (SES) in Alberta, Canada.

**Methods:**

A retrospective population-based cohort study was conducted using linked, province-wide administrative health data for all patients <18 years of age who received a diagnosis of concussion, other specified injuries of head, unspecified injury of head, or post-concussion syndrome between April 1, 2004 and March 31, 2018. Data were geospatially mapped.

**Results:**

Concussion EOCs increased 2.2-fold over the study period, follow-up visits 5.1-fold. Care was increasingly received in physician office (PO) settings. Concussion diagnoses in rural and remote areas occurred in emergency department (ED) settings more often than in metro centres or urban areas (76%/75% vs. 52%/60%). Proportion of concussion diagnoses was positively related to SES and age. Diagnosis and point of care varied geographically.

**Conclusions:**

The shift in care to PO settings, increased incidence of all diagnoses, and the higher use of the ED by some segments of the population all have important implications for appropriate clinical management and the efficient provision of health care for pediatric concussion.

**Impact:**

- This is the first study to use EOC to describe longitudinal trends in incidence and follow-up care for pediatric concussion in relation to age, sex, rurality, point of care, and area-based SES.
- We report increased incidence of concussion in both emergency and outpatient settings and the proportion of diagnoses was positively related to SES and age.
- Patients increasingly received care for concussion in PO over time.
- Geospatial mapping indicated that the incidence of concussion and unspecified injury of head varied geographically and temporally.
- Results have important implications for appropriate clinical management and efficient provision of health care following pediatric concussion.

## Introduction

Millions of children and youth sustain concussions annually in North America.^1^ The incidence of pediatric concussion is likely even higher due to the number that go misdiagnosed, undiagnosed, or unreported.^1^ Compared with adults, children and adolescents are at greater risk for concussion,^2–5^ tend to have more complicated recovery, and require targeted treatment approaches.^2,3,6^ To ensure health services are effective and efficient, a longitudinal population-based understanding of the incidence of pediatric concussion and potential factors impacting health service utilization during defined episodes of care (EOC) is required.

Despite increased awareness and understanding of concussion over the past two decades, most studies have focused on concussions sustained during sport,^7–11^ patients who received care in emergency department (ED) settings,^12–18^ and children over the age of five. Reliance on sport-related and ED-based data underestimates the incidence of pediatric concussion; recent data indicates that approximately one third of pediatric concussions are not sport-related^19^ and that care is increasingly being received in non-ED health care settings.^5,15^ The focus on older children has left a gap in understanding concussion rates and trends among young children, and studies show that concussion is more likely to be diagnosed in children over the age of 10.^20,21^ In addition, as most concussion symptom scales have only been validated in children aged five and older, young children may be misdiagnosed with other non-concussion diagnostic codes.^22^

Furthermore, previous studies often fail to group visits to distinguish a new injury from a follow-up visit and thereby define EOC.^12–15^ This precludes accurate incidence estimates, which may hamper the efficient allocation of resources. Unfortunately, access to care for children and youth can be impeded by significant geographic and socioeconomic barriers,^23–30^ and how and where patients receive care for concussions may differ as a function of geographic differences. Few studies have examined geospatial patterns of concussion incidence, and those that do focused on etiology^17^ or on adults^29^ and are not population-based.^17,29^

This study uses province-wide longitudinal administrative health data to describe trends in the incidence of pediatric concussion and associated factors related to health care utilization in Alberta, Canada. This population-based dataset allowed for tracking of health care utilization within defined EOCs over a 14-year period, enabling the description of trends across diagnoses, time, and diverse demographic, socioeconomic, and geographic characteristics. We hypothesized increasing rates of concussion over time with an accompanying increase in care in outpatient settings relative to ED settings. Secondarily, we hypothesized higher rates of non-specific mild head injuries being diagnosed in younger children, particularly those under the age of five, as well as higher rates among males and those residing in areas of higher socioeconomic status (SES) or in urban areas.^16,17,31^

## Methods

### Study design

A retrospective population-based cohort study was conducted using linked administrative health data from all concussion-related visits in ED, physician offices (PO), and other non-emergency clinics for children under 18 years of age in Alberta, Canada, between April 1, 2004 and March 31, 2018. The province has a centralized health system, and all legal residents have universal access to physician and hospital health care services provided by Alberta Health Services (AHS).^32^ Therefore, these databases serve as a repository of all pediatric concussion-related care across the province (~4.4 million residents, comprising 11% of Canada’s total population). This study followed STROBE reporting guidelines.^33^

**Fig. 1:**
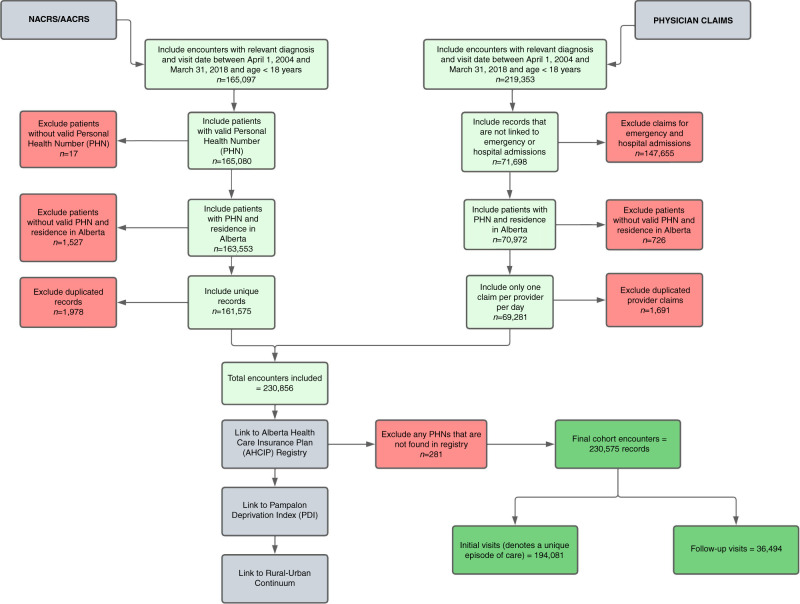
Study cohort creation using the Alberta Ambulatory Care Reporting System (AACRS), National Ambulatory Care Reporting System (NACRS), and physician claims databases. Inclusions are denoted by green boxes and exclusions by red boxes.

Databases included the Alberta Ambulatory Care Reporting System (AACRS) for records April 1, 2004 – March 31, 2010, National Ambulatory Care Reporting System (NACRS) for records April 1, 2010 – March 31, 2018, and physician claims for records April 1, 2004 - March 31, 2018. Unique encoded identifiers and a deterministic linkage approach based on Personal Health Number/Unique Lifetime Identifier (PHN/ULI) and time stamp were used to link the databases (Fig. 1). All cohort identification, data linking, cleaning, and identification was performed by AHS prior to analysis. Ethics approval was obtained from the University of Calgary (REB17-1957_REN3) and administrative approval was obtained from AHS.

### Case Identification

**Fig. 2:**
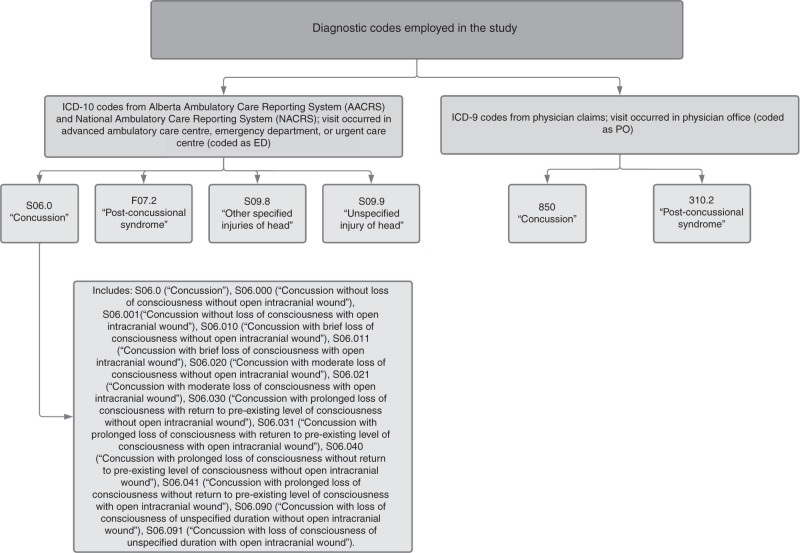
Diagnostic codes employed in the study.

Cases were defined based on the Centers for Disease Control and Prevention (CDC) administrative data definition.^34^ ED visits were captured in AACRS and NACRS and occurred in advanced ambulatory care centres, ED settings, and urgent care centres. These visits were defined as an International Statistical Classification of Diseases and Related Health Problems, 10th Revision (ICD-10) diagnosis of F07.2 (“postconcussional syndrome”), S09.8 (“other specified injuries of head”), S09.9 (“unspecified injury of head”), or one of 13 concussion diagnostic codes (Fig. 2). A maximum of 10 ICD-10 diagnostic codes per visit were recorded and submitted by professional coders. Concussion-related PO visits were captured in physician claims data and took place in outpatient settings, including primary care physicians, medical specialists, and other non-emergency clinics. These visits were defined as an International Statistical Classification of Diseases and Related Health Problems, 9th Revision (ICD-9) diagnosis of 850 (“concussion”) or 310.2 (“postconcussional syndrome”) (Fig. 2). ICD-9 codes are not as specific as ICD-10 codes and are truncated in the physician claims data. As a result, no comparable “unspecified injury of head” or “other specified injuries of head” ICD-9 codes were available for PO visits. A maximum of three ICD-9 codes per visit were recorded and submitted by physicians or their delegates. Excluded records were those with (1) missing or invalid PHN, (2) PHN not found in the Alberta Health Plan registry, (3) patients residing outside of Alberta, (4) duplicated records, (5) invalid postal codes, or (6) missing valid date, diagnosis, or location of health site.

### Definitions and measures

An episode of care (EOC) was defined as an initial index visit plus all subsequent follow-up visits within specific timeframes. A visit was considered within the same EOC if, for an ED visit, no more than 30 days had passed since a previous ED or PO visit, and, for a PO visit, no more than 90 days had passed since a previous ED or PO visit. If no previous visit was found or was outside of the stated timeframe, then the index visit was assigned to a new EOC.

**Fig. 3:**
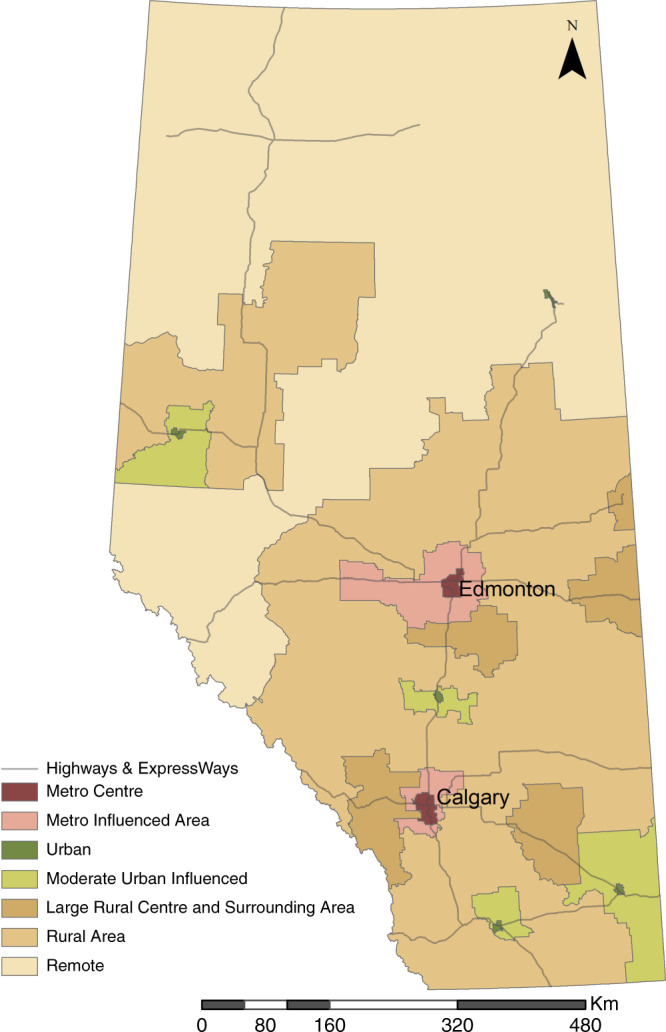
Rural-Urban Continuum in Alberta. Major roadways are indicated.

Community type and location of patient residence was coded based on the Rural-Urban Continuum developed by AHS, which includes the following categories: metro centres, metro-influenced areas, urban areas, moderate urban-influenced areas, large rural centres and surrounding areas, rural areas, and remote areas^35^ (Fig. 3). Patient home addresses are denoted by dissemination area (DA) identifiers, which are used as building blocks to create Local Geographic Areas (LGAs).^35^ The province is divided into 132 LGAs, which are aggregated to 22 zones in the Rural-Urban Continuum (Fig. 3).

Socioeconomic status (SES) was defined by the Pampalon Deprivation Index (PDI). Principal component analysis of several census-derived indicators produces a factor score at the DA level to represent level of deprivation.^36,37^ A lower factor score represents less deprivation (i.e., higher SES) and a higher factor score represents more deprivation (i.e., lower SES).^36,37^ More details on the indicators used to derive the PDI in the specific context of Alberta are provided elsewhere.^37^

### Data analysis

**Fig. 4:**
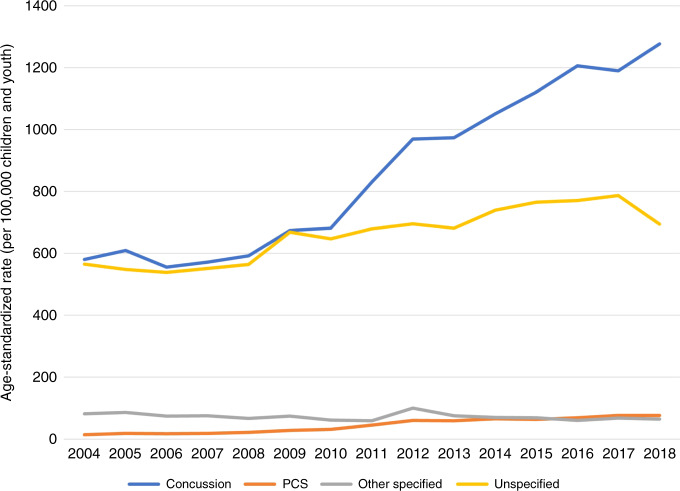
Age-standardized incidence of all pediatric EOC for concussion, PCS, other specified injuries of head, and unspecified injury of head diagnoses occurring in ED and PO settings in Alberta between April 1, 2004 and March 31, 2018, standardized to the 2011 population distribution.

Descriptive analyses were completed on all visits using Stata v. 15.1. Variables included age, sex, location of visit (ED, PO), type of visit (index, follow-up), month of visit, year of visit, SES (PDI), rural-urban continuum category (community size), and diagnosis. The total annual number of visits to ED and PO settings was calculated for each year of the study period by age, sex, community type, and PDI Fig. 4. Crude age-specific rates per 100,000 were calculated using the total number of yearly visits and the estimated Alberta pediatric population size of interest in that year.^38^ Rates were standardized to the 2011 population distribution using direct standardization and Microsoft Excel. Population data were abstracted by AHS from the Alberta Health Care Insurance Plan as a proxy for population counts and included the population of minors per year by community type and by PDI quintile. Population data for age (by year) and sex were retrieved from the Government of Alberta, who received the estimates from Statistics Canada.^39^ Using these populations, incidence was calculated by community type, PDI quintile, age, and sex. Standardized rate ratios (SRR) were calculated between 2004 and 2018 for all diagnoses, visit types, and visit locations. Sensitivity analysis compared annual rates for total number of ED visits for other specified injuries of head and unspecified injury of head (hereafter “other specified” and “unspecified”, respectively) with rates of concussion, to assess whether any increases in concussion diagnosis may reflect a shift away from another head injury diagnosis.

Geospatial analysis was completed on concussion and “unspecified” diagnoses. The geographic boundaries of DAs in Alberta were obtained for 2006,^40^ 2011,^41^ and 2016^42^ census surveys and population estimates by age from Alberta Interactive Health Data Application at the LGA level for 2005, 2011, and 2017.^43^ Diagnoses were summarized at the DA level for 2006, 2011, and 2017 using MS Excel Pivot Table and were then imported into ESRI ArcGIS 10.8 and the data linked to their corresponding DA polygons. DA data were aggregated at the LGA level and then further at the Rural-Urban Continuum level. The number of diagnoses was linked to the corresponding population of children and youth (<18 years of age) and the incidence rates per 100,000 for each LGA and Rural-Urban zone, year, and diagnosis were calculated. Finally, temporal thematic maps of incidence rates were created for each diagnosis to examine temporal and spatial changes.

## Results

The cohort was derived from 384,450 concussion-related encounters across 162,982 unique pediatric patients. In total, 230,575 records were available (Fig. 1). Because an EOC could include multiple visits and a child may have had multiple EOCs during the study, the final sample included 194,081 unique EOC. EOCs could have multiple follow-up visits, and a total of 36,494 follow-up visits occurred during the study period (Tables 1, 2). Of the total EOC, 88% consisted of a single index visit with no follow up (*n* = 168,620). Most patients had a single EOC (*n* = 138,542), 94% of which consisted of a single index visit (*n* = 130,169) and 78% of which began in ED settings (*n* = 107,779).

**Table 1:** Cohort demographics of pediatric concussion, PCS, other specified injuries of head, and unspecified injury of head for initial visits of an EOC to ED and PO settings in Alberta between April 1, 2004 and March 31, 2018.

	Concussion		Post-Concussion Syndrome		Other Specified Injuries of Head	Unspecified Injury of Head	Total Cohort	
	ED	PO	ED	PO	ED	ED	ED	PO
**Total**	101,677		5192		8547	78,665	194,081	
	61,465	40,212	1879	3,313	8547	78,665	150,556	43,525
**Age**								
Median age (years)	13		14		5	4	10	
	13	13	14	14			9	13
**Sex**								
Male	39,223	24,831	1063	1882	5033	46,033	91,352	26,713
Female	22,242	15,381	816	1431	3514	32,632	59,204	16,812
**Community Type of Patient Residence**								
Metro Centre	44,279		2423		3,323	32,525	82,550	
	23,154	21,125	761	1662			59,763	22,787
Metro-Influenced Areas	19,405		1020		992	12,015	33,432	
	11,891	7514	347	673			25,245	8187
Urban	8727		356		688	9011	18,782	
	5205	3522	122	234			15,026	3756
Moderatre Urban-Influenced	2876		131		85	1990	5082	
	1524	1352	43	88			3642	1440
Large Rural Centre and Surrounding Areas	4626		257		424	4254	9561	
	3137	1489	108	149			7923	1638
Rural Areas	19,654		937		2327	15,616	38,534	
	14,982	4672	460	4677			33,385	5149
Remote	2110		68		708	3254	6140	
	1572	538	38	30			5572	568
**Socioeconomic Status (PDI)**^a^								
1 (least deprived/ most advantaged)	18,397		1140		1358	12,736	33,631	
	10,447	7950	342	792			24,883	8,748
2	22,635		1174		1470	15,923	41,202	
	13,292	9343	409	765			31,094	10,108
3	20,362		993		1764	15,935	39,054	
	12,289	8073	374	619			30,362	8,692
4	19,208		929		1629	14,926	36,692	
	12,161	7047	375	554			29,091	7,601
5 (most deprived/ least advantaged)	17,627		783		1881	16,075	36,366	
	11,058	6569	310	473			29,324	7,042

^a^3.6% of the total cohort was missing a PDI quintile (*n* = 8322).

**Table 2:** Follow-up visits for pediatric concussion, post-concussion syndrome, other specified injuries of head, and unspecified injury of head visits to ED or PO in Alberta (2005–2017 fiscal years).

	Concussion		Post-Concussion Syndrome		Other Specified Injuries of Head	Unspecified Injury of Head	Total Cohort	
	ED	PO	ED	PO	ED	ED	ED	PO
**Total**	28,316		5223		247	2707	36,494	
	6508	21,808	1270	3953	248	2707	10,733	25,761
**Age**								
Median age (years)	14		15		8	7	14	
	13	14	14	15			12	14
**Sex**								
Male	4034	13,835	677	2214	144	1,611	6466	16,049
Female	2474	7973	593	1739	104	1,096	4267	9712
**Community Type of Patient Residence**								
Metro Centre	11,686		2,329		86	996	15,097	
	2472	9214	486	1843			4040	11,057
Metro-Influenced Areas	6595		1046		48	619	8308	
	1685	4910	235	811			2587	5721
Urban	2232		335		11	207	2785	
	288	1944	81	254			587	2198
Moderate Urban-Influenced	803		138		2	64	1007	
	173	630	37	101			276	731
Large Rural Centre and Surrounding Areas	1341		229		13	120	1703	
	262	1079	60	169			455	1248
Rural Areas	5128		1060		69	498	6855	
	1459	3669	341	719			2467	4388
Remote	531		86		19	103	739	
	169	362	30	56			321	418
**Socioeconomic Status (PDI)**^a^								
1 (least deprived/ most advantaged)	5313		1142		36	382	6873	
	1006	4307	202	940			1626	5247
2	6885		1168		37	624	8714	
	1698	5,187	265	903			2624	6090
3	5768		1041		57	537	7,403	
	1278	4490	283	758			2155	5248
4	5092		992		35	509	6628	
	1201	3891	258	734			2003	4625
5 (most deprived/ least advantaged)	4347		735		66	542	5690	
	1094	3253	220	515			1922	3768

^a^3.6% of the total cohort was missing a PDI quintile (*n* = 8322).

Of the total EOCs, 52% began with a diagnosis of concussion and 3% with PCS, while nearly 45% received one of the other head injury diagnoses (Table 1). Of the total EOCs, 78% began in ED settings (Table 1). The median age of patients varied across diagnoses and visit type and was higher at index visit when diagnosed with concussion or PCS (Tables 1, 2). Typically, males accounted for a greater proportion of visits for all diagnoses, and most visits occurred in either a metro centre, metro-influenced area, or rural area (Tables 1, 2). Standardized rates of EOC are presented in Table 3 and for follow-up visits in Table 4.

**Table 3:** Standardized rates (per 100,000 children and youth) of pediatric concussion, PCS, other specified injuries of head, and unspecified injury of head for initial visits of an EOC in ED or PO settings in Alberta between April 1, 2004 and March 31, 2018.

	Concussion		Post-Concussion Syndrome		Other Specified Injuries of Head	Unspecified Injury of Head	Total Cohort	
	ED	PO	ED	PO	ED	ED	ED	PO
Overall age-standardized rate (per 100,000) over the study period	859.0		44.2		72.4	659.9	1635.5	
	514.6	344.4	15.5	28.7			1262.4	373.1
Age-standardized rate (per 100,000): 2004	581.0		13.7		81.3	565.6	1241.6	
	359.0	222.0	9.3	4.3			1015.20	226.3
Age-standardized rate (per 100,000): 2018	1277.2		76.7		64.9	694.0	2112.80	
	673.5	603.7	14.9	61.8			1447.30	665.5
Change 2004–2018 (Standardized Rate Ratio)	2.2-fold increase		5.6-fold increase		1.2-fold decrease	1.2-fold increase	1.7-fold increase	
	1.9-fold increase	2.7-fold increase	1.6-fold increase	14.4-fold increase			1.4-fold increase	2.9-fold increase
**Sex (age- and sex-standardized)**								
Male	1047.60		47.8		83.0	751.2	1929.6	
	636.7	410.9	17.2	30.6			1488.1	441.5
Female	660.7		40.3		61.2	563.8	1326.0	
	386.3	274.4	13.7	26.6			1025.0	301.0
**Community Type of Patient Residence (community size-standardized)**								
Metro Centre	336.8		18.2		25.8	249.9	630.7	
	176.9	159.9	5.9	12.2			458.5	172.1
Metro-Influenced Areas	149.3		7.7		7.8	93.2	258.0	
	91.3	58.0	2.7	5.0			195.0	63.0
Urban	68.8		2.8		5.5	71.9	149.0	
	41.3	27.5	1.0	1.8			119.7	29.3
Moderate Urban-Influenced	21.9		1.0		0.7	15.2	38.8	
	11.6	10.3	0.3	0.6			27.8	10.9
Large Rural Centre and Surrounding Areas	26.1		2.0		3.3	33.3	74.7	
	245.5	11.6	0.8	1.2			61.9	12.8
Rural Areas	156.4		7.4		18.4	124.0	306.2	
	119.2	37.2	3.6	3.8			265.2	41.0
Remote	16.8		0.5		5.6	25.7	48.6	
	12.5	4.3	0.3	0.2			44.1	4.5
**Socioeconomic Status (PDI population distribution-standardized)**								
1 (least deprived/most advantaged)	161.8		10.2		11.3	109.0	292.3	
	91.1	70.7	2.8	7.4			214.2	78.1
2	198.0		10.2		12.7	136.4	357.3	
	115.2	82.8	3.4	6.7			267.7	89.5
3	177.7		8.7		15.2	135.7	337.3	
	106.2	71.6	3.3	5.4			260.4	77.0
4	165.9		8.2		13.9	127.3	315.3	
	104.4	61.5	3.2	5.0			248.8	66.5
5 (most deprived/least advantaged)	153.6		6.9		15.9	136.9	313.3	
	95.2	58.4	2.6	4.3			250.6	62.7

Rates are standardized to the 2011 age, sex, community size, or PDI population distribution as indicated.

**Table 4:** Standardized rates (per 100,000 children and youth) of pediatric concussion, PCS, other specified injuries of head, and unspecified injury of head follow-up visits in ED or PO settings in Alberta between April 1, 2004 and March 31, 2018.

	Concussion		Post-Concussion Syndrome		Other Specified Injuries of Head	Unspecified Injury of Head	Total Cohort	
	ED	PO	ED	PO	ED	ED	ED	PO
Overall age-standardized rate (per 100,000) over the study period	246.3		45.3		2.1	22.7	316.4	
	54.6	191.7	10.5	34.8			89.9	226.5
Age-standardized rate (per 100,000): 2004	100.3		13.5		1.8	20.3	135.9	
	37.2	63.1	10.1	3.4			69.4	66.5
Age-standardized rate (per 100,000): 2018	514.5		93.9		1.3	19.2	628.9	
	61.7	452.9	8.3	85.6			90.5	538.5
Change 2004–2018 (Standardized Rate Ratio)	5.1-fold increase		7.0-fold increase		1.4-fold decrease	1.1-fold decrease	4.6-fold increase	
	1.7-fold increase	7.2-fold increase	1.2-fold decrease	25.2-fold increase			1.3-fold increase	8.1-fold increase
**Sex (age- and sex-standardized)**								
Male	299.9		47.8		2.3 (2.3)	26.2 (26.4)	376.4	
	65.4	234.5	10.7	37.1			104.8	271.6
Female	189.9		42.7		1.7 (1.8)	18.5 (18.8)	253.2	
	43.1	146.8	10.2	32.5			73.9	179.3
**Community Type of Patient Residence (community size-standardized)**								
Metro Centre	87.4		17.3		0.7	7.8	113.2	
	19.2	68.2	3.8	13.5			31.5	81.7
Metro-Influenced Areas	50.4		7.8		0.4	4.9	63.5	
	13.5	36.9	1.8	6.0			20.6	42.9
Urban	17.3		2.5		0.1	1.6	21.5	
	2.3	15.0	0.6	1.9			4.6	16.9
Moderate Urban-Influenced	6.0		1.0		0.01	0.5	7.5	
	1.3	4.7	0.3	0.7			2.1	5.4
Large Rural Centre and Surrounding Areas	10.5		1.8		0.1	0.9	13.3	
	2.1	8.4	0.5	1.3			3.6	9.7
Rural Areas	40.9		8.5		0.5	4.7	54.6	
	11.6	29.3	2.7	5.8			19.5	35.1
Remote	4.2		0.7		0.2	0.8	5.9	
	1.3	2.9	0.2	0.5			2.5	3.4
**Socioeconomic Status (PDI population distribution-standardized)**								
1 (least deprived/most advantaged)	48.4		10.5		0.3	3.1	62.3	
	8.6	39.8	1.7	8.8			13.7	48.6
2	61.3		10.6		0.3	5.3	77.5	
	14.4	46.9	2.3	8.3			22.3	55.2
3	51.5		9.1		0.5	4.5	65.6	
	10.8	40.7	2.3	6.8			18.1	47.5
4	45.8		8.7		0.3	4.3	59.1	
	10.3	35.5	2.1	6.6			17.0	42.1
5 (most deprived/least advantaged)	39.3		6.5		0.6	4.6	51.0	
	9.6	29.7	1.8	4.7			16.6	34.4

Rates are standardized to the 2011 age, sex, community size, or PDI population distribution as indicated.

### How and where patients received care

Episodes of care and follow up visits increased over time but differed based on point of care. The average age-standardized incidence rate (ASR) of EOC increased 1.7-fold over the study period, with the most dramatic increase occurring in EOC starting in PO settings. The ASR of follow-up visits increased even more noticeably (4.6-fold), with the increase being more than six times higher in PO (8.1-fold) than in ED settings (1.3-fold) (Table 4). However, the magnitude of increase over time varied by diagnosis and point of care. For concussion diagnoses, the ASR increased 2.2-fold between 2004 and 2018 (Table 3), while the ASR for follow-up increased 5.1-fold (Table 4). The majority of EOCs for concussion began in ED settings (60%), but the ASR for follow-up visits increased more dramatically in PO settings (Tables 1, 3, 4). In fact, care generally shifted to PO over the study period; in 2004, 38% of concussion EOCs began in PO, increasing to 47% by 2018. For follow-up visits, 63% were seen in PO in 2004, versus 88% in 2018. For PCS, follow-up visits exceeded the number of EOC and the ASR of follow-up visits also increased dramatically over the study period, most notably in PO (25.2-fold; (Tables 3, 4). Care for PCS also shifted from ED to PO settings over the study period: in 2004, 31% of index visits for PCS occurred in PO settings, while in 2018 this increased to 80%. For follow-up visits for PCS, 91% occurred in PO in 2018 compared with 75% in 2004. ASR for “other specified” and “unspecified” remained relatively stable, increasing only 1.2-fold over the study period. Follow-up visits coded with either of these diagnoses were rare (Table 2), with 92% of EOCs with those diagnoses consisting of a single visit.

### Age and sex

**Fig. 5:**
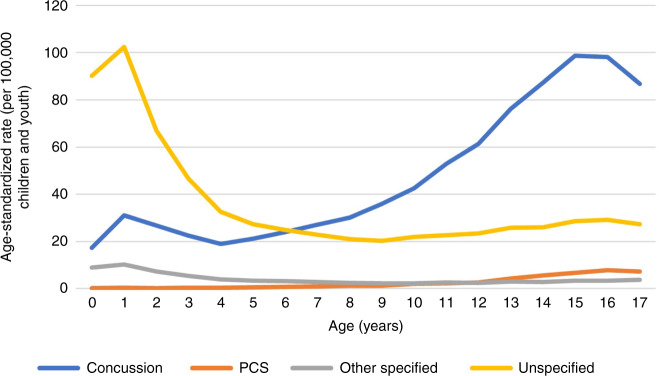
Average age-standardized incidence of pediatric EOC for concussion, PCS, other specified injuries of head, and unspecified injury of head diagnoses occurring in ED and PO settings in Alberta between April 1, 2004 and March 31, 2018, standardized to the 2011 population distribution.

For each year of the study period, the ASR (stratified by sex) were higher for males than females, for both EOC and follow-up visits and in both ED and PO settings. The ASR of concussion was higher in older age groups, while the ASR for other head injury diagnoses was higher in children under age five (Fig. 5). Patients under five comprised only 13% of the total index visits and 5% of the total follow-up visits. Over half of the concussion diagnoses occurred in patients aged 13-17 years. In contrast, other head injury diagnoses occurred primarily in children under age five, including nearly half of both “other specified” (48%) and “unspecified” (50%) visits. The ASR of concussion was lowest in children under one year of age while the ASR of “unspecified” injuries peaked in one-year-old children (Fig. 5). This pattern was also true for “other specified” EOCs. PCS was rare in children under 10 years of age (Fig. 5), who accounted for only 12% of total PCS visits.

### Geographic and socioeconomic variation

The incidence of EOCs and location of care differed by community type. The average community-standardized incidence rate (CSIR) of concussion over the study period was highest in metro centres, which had a CSIR that was 20.0 times higher than in remote areas and 15.4 times higher than in urban-influenced areas (Table 3). The CSIR was 3.2 times higher in ED than PO settings in rural areas but only 1.1 times higher in metro centres (Table 3). In addition, index visits for concussion occurred more often in ED settings in rural and remote areas as compared to metro centres or urban areas (76% and 75% vs. 52% and 60%, respectively) (Table 1).

Location of care for concussion diagnoses also was related to SES – as SES increased, so did the proportion of patients who received care in PO. In other words, the proportion of patients who received care in the ED for concussion increased as SES decreased (Table 1). PDI-standardized incidence rates of concussion were highest in the second, more advantaged, quintile (Table 3). Children and youth from the most advantaged (first) quintile had a PDI-standardized rate of follow-up that was 4.6-fold higher in PO than in the ED, while the rate was only 3.1-fold higher in PO settings for children from the least advantaged (fifth) quintile (Table 4).

Patients in rural and remote areas also received treatment in ED settings for PCS more often than those in metro centres or urban areas, both for index and follow-up visits (Tables 1, 2). Similarly, as SES decreased, the proportion of patients who received care for PCS in the ED increased (Tables 1, 2). The rate of children with EOCs and follow-up visits for PCS were higher in areas of high SES (Tables 3, 4).

Metro centres and rural areas had the highest CSIR of “other specified” and “unspecified” injuries (Table 3), as well as of follow-up (Table 4). These diagnoses also had higher proportions and higher PDI-standardized rates of total EOC among patients from areas of the lowest SES compared with patients from areas of the highest SES (Tables 1, 3).

**Fig. 6:**
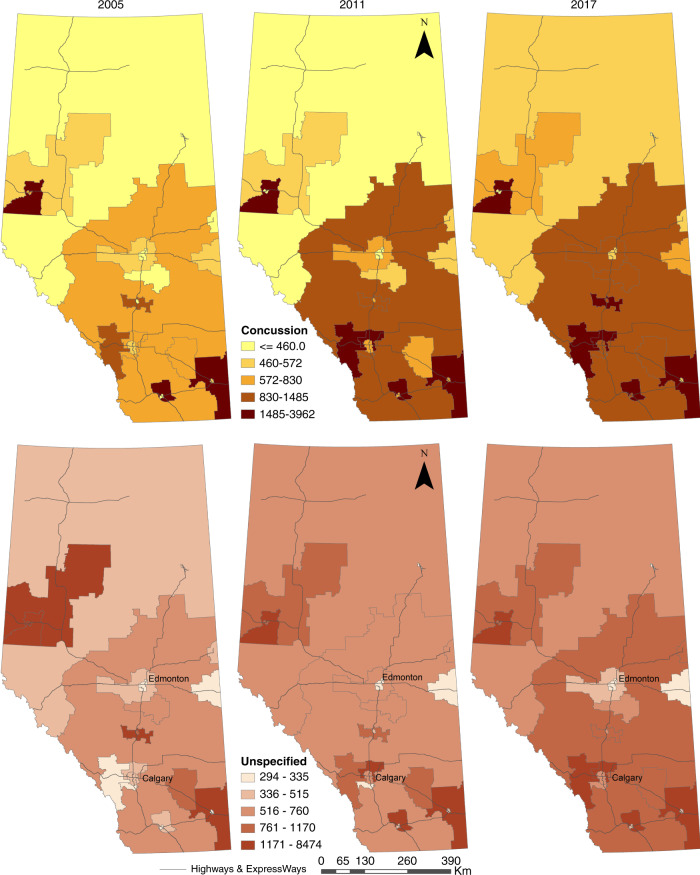
Age-specific incidence of pediatric EOC (rate per 100,000 children and youth) with concussion and unspecified injury of head diagnoses in Alberta in 2005, 2011, and 2017. Major roadways are indicated.

Geospatial analysis showed that increases in the crude rate of concussion and “unspecified” diagnoses were not uniform across community type (Fig. 6). For example, the incidence of concussion EOCs averaged 1,067.3 per 100,000 children and youth in metro-influenced areas (Table 3), but the incidence was higher in and around Calgary than Edmonton (Fig. 6). The same trend is observed for “unspecified” EOCs. Similarly, in rural areas, the average incidence for concussion EOCs was 939.7 per 100,000 and for “unspecified” EOCs was 745.2 per 100,000 (Table 3), but these rates were not consistent across all rural areas. For both diagnoses, large rural centres in the south of the province saw higher rates compared with more central and eastern large rural centres (Fig. 6). The cartographic representation reveals no apparent diagnostic substitution, whereby a low incidence of “unspecified”’ diagnoses is paired with a high incidence of concussion diagnoses and vice versa (Fig. 6). Areas with high (and increasing) incidence are the same for both diagnoses.

## Discussion

This study is one of the first to describe longitudinal trends in the incidence of pediatric concussion and demographic and socioeconomic factors associated with health care utilization for concussion across the entire pediatric age range in a population-based cohort. Our longitudinal approach, use of defined EOC, inclusion of multiple concussion diagnostic codes, inclusion of data from both ED and PO, and geospatial analysis provide novel insights into service use patterns and highlight important socioeconomic and demographic factors associated with pediatric concussion health service use. Concussion EOCs more than doubled and the incidence of age-standardized follow-up visits increased more than five-fold over the 14-year study period. Our results align with previous research indicating that the incidence of pediatric concussion has more than doubled in the past 15 years,^44^ and with studies across North America that have reported increased incidence and health care utilization following concussion in children and youth.^12,14,17,29,31,45,46^

### Shifts in where patients receive care

Concussion care shifted from ED to PO settings over the study period, consistent with recent studies across North America.^13,15,31,47,48^ Clinical guidelines advanced by public health and injury prevention bodies over the past decade suggest that care for concussion should include an initial assessment and diagnosis by a medical professional and regular medical follow up for the duration of symptoms.^49–54^ Our analysis showed increasing rates of follow-up within EOCs, with over three-quarters occurring in outpatient settings. Over the study period follow-up care for all diagnoses increased more noticeably in PO settings, with the increase more than six times that seen in ED settings. Considerable gaps in knowledge have been shown to exist among primary-care providers in managing concussion;^55^ and given our findings, physicians practicing in these settings need to be up-to-date regarding evidence-based guidelines and best practices. Clinical practice guidelines (CPGs) may have played a role in influencing health care utilization related to concussion; however, the rate of follow-up remains well below levels expected if practice were fully aligned with guidelines. Further study to assess the impact of CPGs in follow-up care is needed.

Concussion care also is affected by rurality of patient residence and SES. Consistent with the broader literature pertaining to ED use for non-urgent care,^56,57^ our results indicate higher proportions of patients in rural and remote areas utilizing ED for concussion care. Rural residents are more likely to have lower personal incomes and higher unemployment rates.^58–60^ In Alberta, many of these rural and remote areas fall into medically underserviced areas with fewer providers and services, often forcing patients to seek care in ED settings.^57,61^ As SES increased, the proportion of patients receiving care in the ED decreased. This aligns with recent studies that show people of lower SES use ED services disproportionately more than those of higher SES,^62,63^ regardless of visit urgency.^62,64^ Thus, resources also need to be directed to ensuring ED physicians, especially those in rural and remote areas, are aware of current concussion guidelines. Health system planning aimed at enhancing health services for those with low SES, such as improving PO access, may help reduce the reliance on ED settings.

### Geographic and socioeconomic differences and the potential for unreported concussions

Initial medical evaluation and follow-up care is recommended for all children and youth diagnosed with concussion.^49–54^ Unfortunately, access to care can be impeded by significant geographic and socioeconomic barriers.^23–30^ Our geospatial analysis indicated that the incidence of concussion and “unspecified” diagnoses is uneven across the province. Metro-influenced areas reported the highest rates of concussion and PCS, while rural areas reported the highest rates of “other specified” and “unspecified” EOCs. Regional, institutional, or individual coding differences may have served as a potential confounder and warrant further study. In addition, the reported rate of PCS in areas of high SES was more than double that in those of low SES. Our findings parallel those of a recent study in Ontario^17^ indicating that geographic areas are not homogenous in terms of population, SES, or concussion incidence, highlighting potential variation in access and accessibility. This could be due to financial constraints in regions with lower SES or transportation issues in rural or remote areas, resulting in children and youth from more deprived areas relying on the ED for concussion evaluation and management. We saw higher incidence of concussion EOCs in regions of higher SES when compared with those of lower SES, and particularly in certain areas of the province. While data on mechanism were not available in this study, higher SES has been associated with increased involvement in organized sports for children and youth^65^ and sports and recreation-related activities are the primary source of concussions among older children and youth.^19^ Thus, differences in rates of concussion by region and SES may partly reflect differential access to and participation in organized sports. But the possibility also exists that such differences reflect less access to care in lower SES and rural settings, resulting in underreporting of concussions.

### Diagnostic differences and the potential misdiagnosis of concussion

Younger children have higher rates of mild head injuries that are not diagnosed as concussion. The “unspecified” diagnosis is often used when altered mental status is not documented,^66^ and because young children have limited communication abilities, this can be difficult to detect.^67^ We saw an increase in diagnosis of other mild head injuries over the study period, with rates highest in patients under five years of age. At the same time, patients under five had the lowest rates of concussion. This is consistent with a recent study in Quebec that found children under four had nearly double the rate of mild head injury diagnoses other than concussion when compared with older groups of children and youth.^68^ Thus, some of the children under five in our study may have been misdiagnosed. On the other hand, we must acknowledge the potential inclusion of other unrelated, non-concussive head injury conditions in our data (e.g., lacerations) and the possibility that the true rate of concussions is misrepresented by including these non-specific diagnoses. Future work in this area is recommended to determine whether these other injuries are in fact concussions in younger children. In the meantime, increasing awareness among providers and parents that these other head injuries can in fact be concussions in infants and younger children may help to ensure appropriate care for these patients. In addition, further development of clinical guidelines and symptom assessment for concussion management in young children is needed to ensure accurate diagnosis and treatment.

## Limitations and conclusion

Our findings reflect trends in child and youth concussion follow-up care in Alberta, and may not generalize to other jurisdictions due to local factors that may facilitate or impede access to health care. However, as all Canadian provinces have universal health care systems similar to that in Alberta, we would expect trends in other provinces to be similar. In addition, administrative health data have inherent challenges. Data quality and validity are unknown. Validation of concussion codes was not performed separately in our dataset; therefore, miscoding, non-specific diagnoses, and overrepresentation of certain diagnoses may have resulted in inaccurate diagnostic coding, skewing our incidence estimates.^69^ However, studies report that the quality of administrative data in Canada is high^69–71^ and our inclusion of other mild head injury diagnoses helped to capture potential misdiagnoses. EOCs could be misclassified in some instances because claims data lacked time stamps, preventing accurate classification of a PO and ED visit occurring in the same day. Additionally, based on our definition of EOC, separate concussions that occurred close in time (i.e., within the definition of EOC) would be considered as follow-up visits and not new injuries. In addition, care provided in non-ED or PO settings or by allied health practitioners or sports trainers, who do not bill directly through the public health care system (e.g., chiropractors, physiotherapists, neuropsychologists or other specialists), was not represented in the administrative data.

We explored head injuries other than concussion (“other specified” and “unspecified” injuries) only for ED visits, because similar codes were not available for PO visits. We initially considered several additional ICD-9 codes for inclusion, but due to the nature of the claims data, ICD-9 codes for other head injuries were not as specific as ICD-10 codes or were truncated, precluding the isolation of head injuries. We examined the proportion of claims related to these potentially comparable diagnostic codes and found that they were used for less than 10% of total PO visits; this compares to almost 58% of total ED visits being diagnosed with “unspecified” and “other specified” injuries. Thus, the omission of these codes for PO visits seems unlikely to have had a major influence on our results.

LGAs were aggregated into the regions of the Rural-Urban Continuum; however, some small rural areas have missing data. In our case, 3.6% of all records (*n* = 8,322) were missing a PDI quintile. This is likely due to small populations, as accurate calculation of the PDI is not possible where a DA has a very small population or a missing or invalid postal code.^37^ This has implications for the interpretation of SES in these communities, as the PDI may not accurately reflect the true proportion of patients. In addition, First Nations, homeless, and other hard to reach populations are under‑represented due to census non‑response. These groups tend to be of lower SES, which potentially underestimates the population in these quintiles.^37^ Furthermore, postal codes in rural and remote areas also tend to cover larger geographical areas than postal codes from other areas, resulting in greater heterogeneity of SES among residents.^72^ There is also risk of ecological fallacy with applying group SES indicators to inferences at the individual level. As such, future studies should use individual estimates of SES rather than an aggregate area-based socioeconomic indicator.

Despite these limitations, we saw concussion-related EOC nearly double between 2004 and 2018 in Alberta, and follow-up visits more than quadruple. Geospatial and temporal analysis of concussion-related diagnoses across the province indicated shifting trends in where patients receive care and which patients may be more likely to receive initial or follow-up care. More specifically, the shift in care from ED to PO settings, increased incidence of all head injury diagnoses in the population, and the higher use of the ED by certain segments of the population all have important implications for appropriate clinical management and efficient provision of health care for pediatric concussion. Using these results, prevention, awareness, and management efforts can be targeted to optimize health care utilization and promote healthy recovery from concussion.

## Supplementary information

STROBE Checklist_R2

## Data Availability

The data used in this study were collected from multiple sources. Databases included the Alberta Ambulatory Care Reporting System (AACRS) for records April 1, 2004 – March 31, 2010, National Ambulatory Care Reporting System (NACRS) for records April 1, 2010 – March 31, 2018, and physician claims for records April 1, 2004 - March 31, 2018. The data are restricted and permissions to access must be obtained from Alberta Health Services (AHS). Population data for age (by year) and sex were retrieved from the Government of Alberta, who received the estimates from Statistics Canada.^39^ Population data for community size and PDI quintile were abstracted by AHS from the Alberta Health Care Insurance Plan as a proxy for population counts and permission for access must be obtained from AHS. In geospatial analysis, the geographic boundaries of DAs in Alberta were obtained from Statistics Canada for 2006,^40^ 2011,^41^ and 2016^42^ and population estimates by age were retrieved from Alberta Interactive Health Data Application at the LGA level for 2005, 2011, and 2017.^43^
